# Mitochondrial hydrogen peroxide production by pyruvate dehydrogenase and α-ketoglutarate dehydrogenase in oxidative eustress and oxidative distress

**DOI:** 10.1016/j.jbc.2023.105399

**Published:** 2023-10-28

**Authors:** Olivia Chalifoux, Ben Faerman, Ryan J. Mailloux

**Affiliations:** Faculty of Agricultural and Environmental Sciences, The School of Human Nutrition, McGill University, Ste.-Anne-de-Bellevue, Quebec, Canada

**Keywords:** pyruvate dehydrogenase, α-ketoglutarate dehydrogenase, oxidative eustress, oxidative distress, mitochondria, hydrogen peroxide, fatty liver disease, redox signaling

## Abstract

Pyruvate dehydrogenase (PDH) and α-ketoglutarate dehydrogenase (KGDH) are vital entry points for monosaccharides and amino acids into the Krebs cycle and thus integral for mitochondrial bioenergetics. Both complexes produce mitochondrial hydrogen peroxide (mH_2_O_2_) and are deactivated by electrophiles. Here, we provide an update on the role of PDH and KGDH in mitochondrial redox balance and their function in facilitating metabolic reprogramming for the propagation of oxidative eustress signals in hepatocytes and how defects in these pathways can cause liver diseases. PDH and KGDH are known to account for ∼45% of the total mH_2_O_2_ formed by mitochondria and display rates of production several-fold higher than the canonical source complex I. This mH_2_O_2_ can also be formed by reverse electron transfer (RET) *in vivo*, which has been linked to metabolic dysfunctions that occur in pathogenesis. However, the controlled emission of mH_2_O_2_ from PDH and KGDH has been proposed to be fundamental for oxidative eustress signal propagation in several cellular contexts. Modification of PDH and KGDH with protein S-glutathionylation (PSSG) and S-nitrosylation (PSNO) adducts serves as a feedback inhibitor for mH_2_O_2_ production in response to glutathione (GSH) pool oxidation. PSSG and PSNO adduct formation also reprogram the Krebs cycle to generate metabolites vital for interorganelle and intercellular signaling. Defects in the redox modification of PDH and KGDH cause the over generation of mH_2_O_2_, resulting in oxidative distress and metabolic dysfunction-associated fatty liver disease (MAFLD). In aggregate, PDH and KGDH are essential platforms for emitting and receiving oxidative eustress signals.

Metabolic dysfunction associated fatty liver disease (MAFLD) affects 25% of North Americans and occurs in 35% of people worldwide (1, 2, 3, 4). Its surge is due to the prevalence of obesity, type 2 diabetes mellitus, metabolic syndrome, and exposure to poor diets, environmental toxins, and xenobiotics (1, 2, 3, 4). MAFLD has a wide spectrum of hepatic manifestations ranging from simple steatosis to more severe forms characterized by cell death, necrosis, fibrosis, inflammation, non-alcoholic steatohepatitis (NASH), and cirrhosis, which are risk factors for the development of hepatocellular carcinoma (HCC) (1, 4). Despite its increased frequency, there are still very few effective curative pharmacological approaches for MAFLD. This is because many MAFLD cases are asymptomatic, making its manifestation difficult to detect until the more advanced stages of the disease (5). MAFLD also has a higher preponderance in men when compared to pre-menopausal women but surges post-menopause (2, 6). Abnormal mitochondrial function and redox imbalance causing oxidative distress have been identified as common distinguishing features for the progression of MAFLD (3, 5, 7). The use of rodent models for MAFLD delineated mitochondrial hydrogen peroxide (mH_2_O_2_) accumulation and the induction of oxidative distress as an early event in the development of MAFLD (8, 9, 10). Sex dimorphisms in MAFLD are associated with superior mitochondrial redox poise, mH_2_O_2_ budgeting, and fuel metabolism in female rodents and pre-menopausal women (11). MAFLD is accelerated in ovariectomized rodents and post-menopausal women which is related to defective mitochondria and the induction of oxidative distress (2, 12). This underscores the importance of mH_2_O_2_ budgeting in optimal hepatic health.

MAFLD can be prevented or even reversed by thiamine supplementation, a cofactor required for PDH and KGDH activity (13). PDH and KGDH activation have been implicated in preventing MAFLD by improving glucose metabolism and promoting better cellular redox poise (14). Together, this implies maintenance of optimal PDH and KGDH activity could be a useful tool in treating or preventing MAFLD. The keto acid dehydrogenases PDH and KGDH occupy pivotal positions in mitochondrial bioenergetics as both enzymes connect monosaccharide and amino acid catabolism to the electron transport chain (ETC) and oxidative phosphorylation (OxPhos) (Fig. 1). PDH catalyzes the oxidative decarboxylation of pyruvate formed by the glycolytic metabolism of monosaccharides, producing acetyl-CoA and the electron carrier NADH (Fig. 1) (15, 16, 17, 18). Acetyl-CoA then condenses with oxaloacetate through a Claisen condensation reaction driven by citrate synthase to prime the Krebs cycle. Ala, Gly, Cys, Ser, and Thr also generate pyruvate through transamination reactions, making PDH important for the intake of some amino acids into catabolic pathways (19). KGDH is the fourth enzyme in the Krebs cycle and couples the oxidation of α-ketoglutarate to the generation of CO_2_, NADH, and succinyl-CoA (Fig. 1) (20, 21). The succinyl-CoA is then metabolized further by the Krebs cycle. Glutamate is a major source and sink for amines in mammalian cells. Its production occurs through the transamination of α-ketoglutarate by aminotransferases (22, 23). Glutamate catabolism can also be facilitated by glutamate dehydrogenase, which couples its oxidative deamination to the formation of NAD(P)H and α-ketoglutarate (23, 24). Glutamate fuels cancer cell proliferation, survival, and metastasis, making this pathway an important therapeutic target for cancer treatment (24). Glutamate is also formed by the transamination of Ala, Gly, Cys, Ser, and Thr as well, linking pyruvate metabolism to the α-ketoglutarate pool. Collectively, KGDH is integral for the metabolism and provision of amino acids for cell processes.

**Figure 1:**
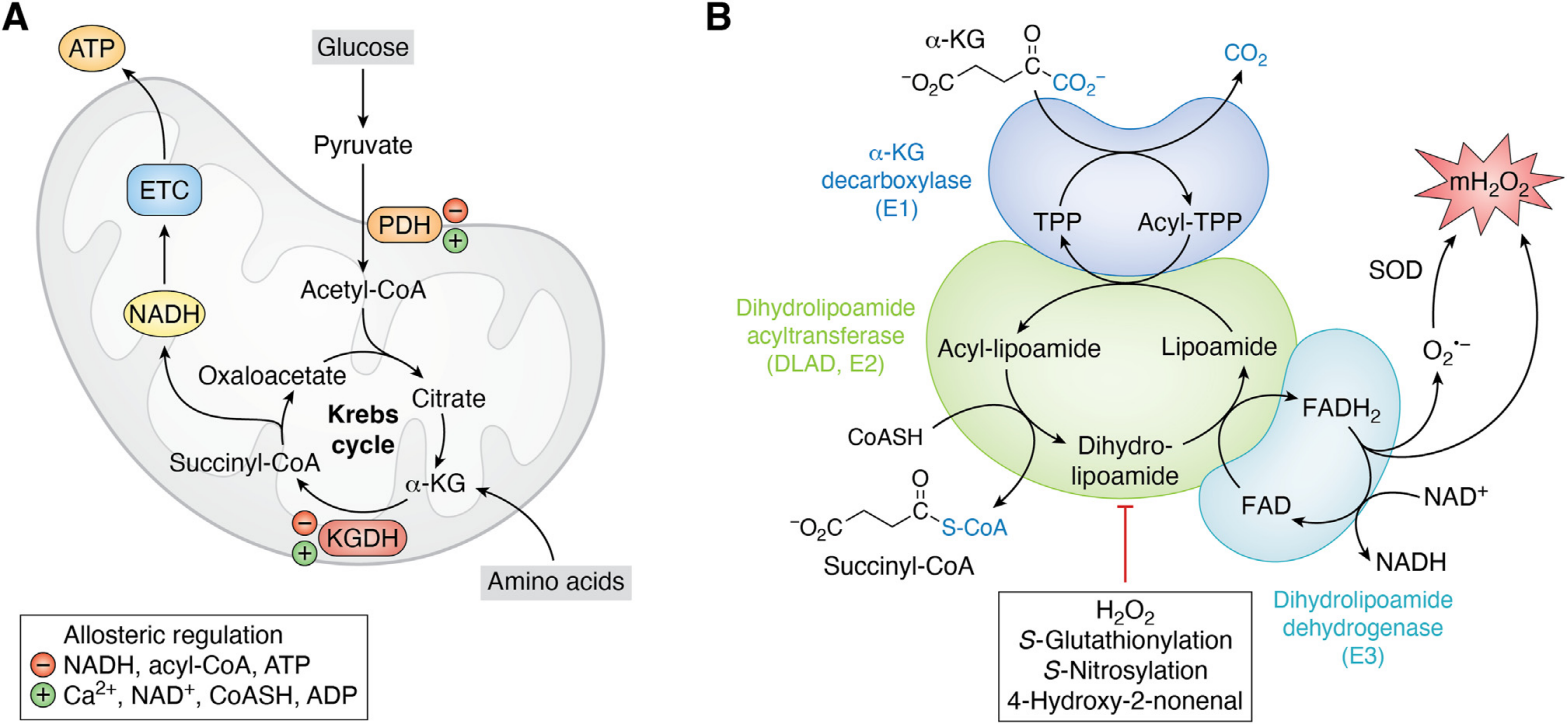
**The entry of carbon into the Krebs cycle through PDH and KGDH, the catalytic cycle of the α-ketoacid dehydrogenases, and the sites for redox regulation and mH**_**2**_**O**_**2**_**generation.***A*, the entry of monosaccharides (glucose) and amino acids into mitochondria and the Krebs cycle through pyruvate dehydrogenase (PDH) and α-ketoglutarate dehydrogenase (KGDH). Acetyl-CoA and succinyl-CoA formed by PDH and KGDH are oxidized further by the rotation of the Krebs cycle, producing the electron carrier NADH which is metabolized by the electron transport chain (ETC) to generate ATP by oxidative phosphorylation (OxPhos). PDH and KGDH catalyze high energy reactions in the Krebs cycle and are thus important sites for allosteric activation and deactivation (denoted by *red* × or *green triangle*). *B*, the catalytic pathway for the keto acid dehydrogenases using KGDH as an example. α-ketoglutarate is decarboxylated on the C1 position (in *blue*) by α-ketoglutarate decarboxylase (E1), transferring the succinyl group to thiamine pyrophosphate (TPP). The dihydrolipoamide succinyltransferase (or acyltransferase: E2, DLAT) catalyzes the formation of a high-energy thioester bond *via* the transfer of the succinyl moiety from TPP to coenzyme A (CoASH). The dihydrolipoamide is then oxidized by the dehydrogenase activity of the E3 subunit (dihydrolipoamide dehydrogenase or DLD), reducing FAD to FADH_2_ and then producing NADH. Electrons leak from the FAD through side reactions that generate semiflavin radicals, flavin hydroperoxides, and oxy-flavin radicals, which form mH_2_O_2_. Note that the FAD can generate both mitochondrial superoxide (mO_2_^•−^) and mitochondrial (mH_2_O_2_), reactions that depend on the redox state of the flavin and its interactions with molecular oxygen (O_2_). The mO_2_^•−^ is dismutated to mH_2_O_2_ by superoxide dismutase (SOD; intermembrane space = SOD1, matrix = SOD2). The E2 dihydrolipoamide is a target for redox modifications by mH_2_O_2_ and various electrophiles and soft acid metals (*e.g.*, arsenic) which block the activity of the enzyme but also inhibit mH_2_O_2_ generation. The figure was generated with Biorender Software (Agreement Number: NR25TFIEU4).

Two decades of research building on key initial studies have revealed that PDH and KGDH are important sources and sinks for mH_2_O_2_ (25, 26). The FAD center in the E3 forms mH_2_O_2_ during forward electron transfer (FET) after α-ketoacid decarboxylation (Fig. 1) (26, 27). NADH also supplies electrons for mH_2_O_2_ production by PDH and KGDH through reverse electron transfer (RET) (26, 28). There is also evidence implicating the E1 subunit in mitochondrial superoxide (mO_2_^•−^) generation through a thiamine radical intermediate (29, 30). PDH and KGDH are inhibited by mH_2_O_2_ through oxidation of vicinal thiols on the dihydrolipoamide (31). It is now understood PDH and KGDH exhibit rates of mH_2_O_2_ generation greater than classical sources like complex I in several tissues. Furthermore, it was revealed this mH_2_O_2_ generation can be decelerated by redox signals such as PSSG and PSNO (32, 33). Both modifications deactivate mH_2_O_2_ to prevent oxidative distress and desensitize mitochondrial eustress signals in liver tissue and macrophages. Redox modification of the dihydrolipoamide also reprograms the Krebs cycle for coordination of cell functions. In the sections below, we elaborate on the central importance of PDH and KGDH in facilitating mitochondrial oxidative eustress signals and how disruption of the redox state of both enzymes leads to oxidative distress and the manifestation of MAFLD. Further, we propose these two enzymes may serve as novel therapeutic targets for the treatment of hepatic diseases like MAFLD, NASH, and HCC.

## Structure and catalytic cycle of PDH and KGDH

PDH and KGDH are multimer complexes composed of multiple copies of three subunits: E1 (pyruvate or α-ketoglutarate decarboxylase), E2 (dihydrolipoamide acyl transferase; DLAT), and E3 (dihydrolipoamide dehydrogenase; DLD) (34). PDH and KGDH are part of the keto acid dehydrogenase family of enzymes, which are unique because they rely on many co-factors and prosthetic groups for their activity (35). The enzymes require thiamine pyrophosphate (TPP: E1 subunit), CoASH and lipoic acid (E2 subunit), and FAD and NAD (E3 subunit). These factors and subunits work in tandem to produce acyl-CoAs and NADH following the oxidative decarboxylation of substrates (35). Uniquely, only these enzymes require lipoate for their activity, which is synthesized in the matrix of mitochondria by *de novo* lipogenesis (36, 37). The pathway relies on the biosynthesis of octanoyl-ACP, its transsulfuration by S-adenosyl methionine, and then its transfer to the E2 subunit of the keto acid dehydrogenase (36, 38). This makes keto acid dehydrogenases highly unique because their activity depends on the posttranslational modification of the E2 subunit by a product of mitochondrial lipogenesis, which can then be targeted for redox regulation.

Mammalian PDH has a stoichiometry of 40:40:20 for the E1, E2, and E3 subunits forming a ∼9.5 MDa multisubunit holoenzyme (39). In contrast, it is predicted that KGDH is ∼3.2 MDa and comprised of 12 E1 and 12 E3 subunits surrounding a 24-mer E2 (40). Additional structural components for PDH include dihydrolipoamide dehydrogenase-binding protein (E3BP), which forms part of the structural core of PDH (41). PDH is also associated with kinase (PDK) and phosphatase (PDP) (42). Cells contain several PDK isoforms (PDK1–4), which display tissue-specific expression (42). PDK inhibits PDH in response to hypoxia, high rates of fatty acid oxidation, and nutrient deprivation and starvation (43). Targeting PDK has high therapeutic potential for the treatment of several pathologies including diabetic cardiomyopathy, cancer, thrombosis, cholestasis, and many others (43, 44, 45). Notably, PDK2 is deactivated by reversible oxidation of cys-45 and cys-392, providing an extra layer of redox regulation for PDH (46). By contrast, KGDH harbors an adaptor protein called KGD4 that is essential for the assembly of the E1 and E2 with the E3 subunits (47). Of note, α-ketoglutarate metabolism can also produce an oncometabolite called 2-hydroxyglutarate. Thus, the targeted disruption of its formation has high therapeutic potential for cancer treatment as well (48). Compounds like the dihydrolipoamide analog CPI-613 (Devimistat) have also been developed for onco-treatment as it disrupts PDH and KGDH metabolism, thereby deactivating metabolic programs that promotes carcinogenesis (49). Our group has since applied CPI-613 for the study of mH_2_O_2_ formed by PDH and KGDH (50). It has proven to be a valuable tool in the study of mitochondrial redox biology.

PDH and KGDH share the same basic catalytic mechanism (Fig. 1). The enzyme cycle begins with the binding of the α-ketoacid to the E1 subunit. This triggers the decarboxylase activity of the E1 subunit, releasing CO_2_ from the C_1_ position of either α-keto acid and the acylation of TPP (51). The transferase activity of the E2 subunit acylates the covalently bound vicinal thiol containing dihydrolipoamide, producing an acyl-lipoyllysine intermediate (31). The acyl-group is then *trans*-esterified to CoA to form acetyl-CoA or succinyl-CoA (52). The oxidation-reduction event on the lipoyl vicinal thiols drives the formation of the high-energy thioester bond in the acyl-CoAs (52). The final step of the cycle is catalyzed by the dehydrogenase (E3) subunit. The reduced lipoyllysine is oxidized by the E3 subunit, transferring electrons to FAD to regenerate the disulfide required for E2 catalysis. This forms FADH_2_ which is then oxidized to form NADH (19). The FAD also generates a mixture of mO_2_^•^ and mH_2_O_2_, which depends on the oxidation state of the flavin (Fig. 1) (53). The acetyl-CoA and succinyl-CoA are used to power subsequent oxidations in the Krebs cycle, while NADH fuels OxPhos. PDH and KGDH catalyze the irreversible conversion of pyruvate or α-ketoglutarate to an acyl-CoA and are thus vital points for metabolic regulation. Both are activated by calcium signaling from the endoplasmic reticulum, which leaks calcium into mitochondria at mitochondria-associated membrane (MAM) contact sites (Fig. 1) (54). The complexes are also subjected to allosteric activation and deactivation by various metabolites and their metabolic products. Acetyl-CoA or succinyl-CoA inhibits PDH and KGDH, respectively, and both enzymes are also modulated by the NADH/NAD^+^ and ATP/ADP ratios and the availability of CoASH relative to its acylated form (Fig. 1). Deactivation of PDH and KGDH leads to pyruvate and α-ketoglutarate accumulation, which serve as intercellular signaling molecules that activate G-protein coupled receptors (GPR) (55, 56). Discussing the catalytic mechanism and allosteric regulation of KGDH and PDH is vital since both affect their function in oxidative eustress signaling and the induction of oxidative distress during cell stress. Activation and deactivation of both enzymes also reprograms the Krebs cycle for the accumulation of metabolites for cell communication and anabolism.

## PDH and KGDH as sources for mH_2_O_2_

### Oxidative eustress and oxidative distress; PDH and KGDH as major mH_2_O_2_ suppliers in mitochondria

**Figure 2:**
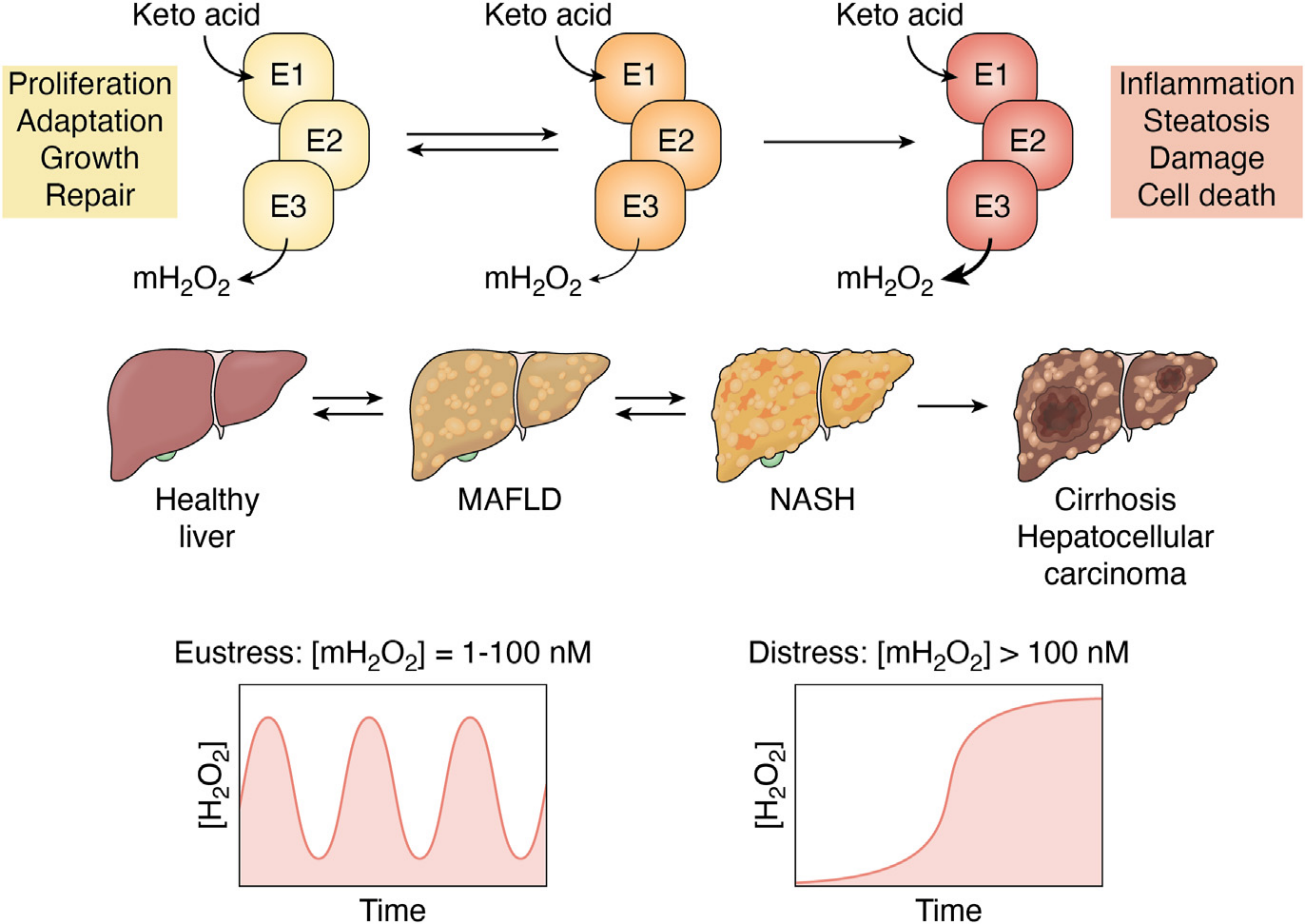
**Oxidative eustress and oxidative distress and the role of pyruvate dehydrogenase (PDH) and α-ketoglutarate dehydrogenase (KGDH) in supplying mitochondrial (mH**_**2**_**O**_**2**_**) for optimal liver health.** Spatio-temporal control over mH_2_O_2_ generation triggers oxidative eustress pathways (*left*) which activate proliferation, adaptation, growth, and repair cascades required for the restoration of normal hepatic function and maintaining optimal liver health. The mH_2_O_2_ levels oscillate in the 1 to 100 nM range, which is achieved, in part, through the regulation of its production through activation and inhibition of PDH and KGDH. Defects in the regulation of mH_2_O_2_ generation by PDH and KGDH result in its sustained overproduction. This sustained overgeneration of mH_2_O_2_ is due to factors that promote mitochondrial dysfunction (*e.g.*, toxins or poor nutrition). This promotes mH_2_O_2_ accumulation, triggering oxidative distress. This oxidative distress (*right*) is characterized by defective oxidative eustress signals, over-oxidation of antioxidant defenses, and cell damage and death. These effects result in inflammation, hepatic ballooning, and metabolic dysfunction causing irreversible liver damage (cirrhosis) and the development of hepatocellular carcinoma. Note that the early stages of MAFLD is reversible. However, sustained oxidative distress induced by the continued accumulation of mH_2_O_2_ causing progressive cell damage and perturbed eustress leads to irreversible liver disease. The figure was generated with Biorender Software (Agreement number: KN25TFIEZ2).

The term “*oxidative stress*” refers to an imbalance in oxidants and antioxidants in favor of the former, which can lead to cell damage and death (57). *Oxidative stress* was first coined by Professor Helmut Sies, who recently collaborated with Professor Dean Jones to develop new terms, “*oxidative eustress*” and “*oxidative distress*”, to extend this original definition (58, 59). These two terms are highly useful since both distinguish between a positive oxidative stress that triggers changes in cell behavior in response to stimuli and a negative one that leads to macromolecular damage, tissue dysfunction, disease, and death (Fig. 2) (58, 59). Importantly, oxidative eustress is a signal that occurs when mH_2_O_2_ is in the low-to-high nM range (*e.g.*, 1–100 nM) (58, 59, 60, 61, 62, 63). These signals are propagated through mH_2_O_2_ generation, which can be generated directly by oxidoreductases and dehydrogenases (*e.g.*, PDH or KGDH) or indirectly through O_2_^•^ production followed by its rapid dismutation by superoxide dismutase (SOD, SOD1 = cytoplasm and intermembrane space, SOD2 = matrix) (64, 65) (Figs. 1 and 2). There are 42 O_2_^•^ and/or H_2_O_2_ generators in mammalian cells and 16 of these sources are housed in mitochondria (58, 59, 60, 61, 62, 63). The “*eu*” in *eustress* is Greek for *good* and thus its incorporation into the term is appropriate given the mH_2_O_2_ is inducing a positive cell outcome. By contrast, oxidative distress is triggered under conditions that promote the accumulation of mH_2_O_2_ and other oxidants (60, 61, 62, 63) (Fig. 2). This occurs at >100 nM mH_2_O_2_ and results in dysfunctional eustress signaling through non-specific and irreversible protein oxidations, culminating with macromolecular damage and cell death (58, 66) (Fig. 2). The activation of oxidative eustress or distress depends on the redox stress signaling threshold (RST) of the organism (67). In the case of the liver, short and controlled bursts in mH_2_O_2_ production activates cell proliferation and adaptive responses after injury, making oxidative eustress signals important for optimal hepatic health (68) (Fig. 2). However, imbalanced mH_2_O_2_ generation due to loss of control over its formation and its subsequent accumulation causes dysfunctional redox signaling, leading to oxidative distress and the manifestation of metabolic dysfunction-associated fatty liver disease (MAFLD) (3) (Fig. 2).

Both PDH and KGDH are targets for oxidative distress (26, 28). PDH and KGDH were both found to be mH_2_O_2_ generators in rat brain mitochondria and synaptosomes 2 decades ago (26, 28). At the time, it was hypothesized that mH_2_O_2_ generation by both enzymes was linked to oxidative distress and pathogenesis (26, 69, 70, 71). Since then, a significant amount of data has been generated demonstrating both PDH and KGDH are important sources of mH_2_O_2_. Empirical evidence collected by Professor Martin Brand’s group has shown KGDH and PDH produce 8× and 4× more mH_2_O_2_ in rat muscle mitochondria when compared to complex I (72, 73). The same group also described how this production depends on substrate supply, with Krebs cycle linked substrates activating mH_2_O_2_ generation by PDH and KGDH whereas nutrients that donate electrons directly to the ETC do not induce production by either enzyme (74). However, as described below, Horvath *et al.* (75) recently challenged this convention by showing substrates that are oxidized directly by the ETC can drive mH_2_O_2_ generation by reverse electron transfer (RET) to KGDH through complex I. PDH and KGDH account for ∼12% and ∼35%, respectively, of the total mH_2_O_2_ produced by liver mitochondria (50, 76). In addition, PDH and KGDH display much higher rates of mH_2_O_2_ production when compared to other “unconventional” sources like proline dehydrogenase (PRODH) and *sn*-glycerol-3-phosphate dehydrogenase (GPD) (76). Notably, PDH is also a vital source of mH_2_O_2_ in permeabilized muscle fibers of mouse, rat, and human origin (77, 78). In this context, it was reported the PDH works in tandem with transhydrogenase to maintain mitochondrial redox buffering capacity, which protects mice from diet-induced obesity (77). Similar findings were made with KGDH and transhydrogenase in cardiac tissue, suggesting sustained and controlled mH_2_O_2_ generation and its communication with transhydrogenase is required for heart function (79). Finally, a recent study showed PDH is an important mH_2_O_2_ generator in macrophages (32). These findings also apply to concepts in oxidative eustress signaling. Indeed, complex III, and to a lesser extent complex I, are often viewed as the sole mH_2_O_2_ sources in mitochondrial redox communication. The discovery that PDH and KGDH also produce large quantities of mH_2_O_2_ shows both enzymes are likely to be part of the mitochondrial oxidative eustress signaling platform too, and may be more important than complexes I and III in cell redox signaling under certain physiological conditions.

### The effect of sex on PDH and KGDH and the manifestation of MAFLD

Another important observation is the sex effect on mH_2_O_2_ generation by PDH and KGDH. Indeed, PDH and KGDH generate ∼3-fold and ∼6-fold more mH_2_O_2_ in male mouse liver mitochondria when compared to females (80). These sex dimorphic effects mean mH_2_O_2_ signals are quite different in males and females as redox tone serves as a vital interface for coordinating cell behaviors in response to stimuli. It also accounts for some important sex differences in the sustained overproduction of mH_2_O_2_ in the pathogenesis of liver diseases. Mitochondria have great therapeutic potential for the development of sex-specific therapies in the treatment of pathologies (11). For example, abnormal mitochondrial function and redox buffering are hallmarks of the development of MAFLD (3). MAFLD is a broad spectrum of hepatic disorders ranging from simple steatosis to more severe forms like cirrhosis and hepatocellular carcinoma (1). Importantly, the manifestation of MAFLD is more prominent in male rodent models and men (1). However, rates in women (and female rodent models) increase significantly with the onset of menopause (2). Oxidative distress due to higher-than-normal mH_2_O_2_ generation for a prolonged period has been linked to the pathogenesis of MAFLD and the onset of its more severe forms (Fig. 2) (3). In fact, over generation of mH_2_O_2_ with no detectable presence of defects in mitochondria is an early event in MAFLD progression (8). A recent report found targeted suppression of mH_2_O_2_ by the ETC prevented MAFLD and promoted hepatic regeneration after injury from poor nutrition (81). This was achieved using S1QEL1.719, which has been documented to selectively inhibit mH_2_O_2_ generation by complex I (81). Notably, PDH and KGDH are more potent mH_2_O_2_ sources than complex I in hepatocytes. Thus, targeted suppression of mH_2_O_2_ generation by PDH and KGDH and/or restoration of eustress signals initiated by both enzymes may be beneficial for hepatic regeneration after injury. The sex differences in mH_2_O_2_ generation by PDH and KGDH also suggests treatment can be tailored to both men and women at different ages.

There is strong evidence to support the role of KGDH, and to a lesser extent PDH, in serving as major mH_2_O_2_ sources in this context *in vivo*. For instance, as discussed above, PDH and KGDH can cause oxidative distress through the sustained higher-than-normal generation of mH_2_O_2_, which can cause MAFLD and the development of its more severe forms. However, the controlled generation of mH_2_O_2_ is vital for oxidative eustress signaling in hepatic regeneration in response to injury (68). Thus, understanding how mitochondria budget mH_2_O_2_ for communication is important. Conventional substrates that prime the Krebs cycle like glutamate or pyruvate (supplied with malate) have been found to drive high rates of mH_2_O_2_ generation by PDH and KGDH (26). In this context, our group was successful in demonstrating that PDH and KGDH exhibit high rates of mH_2_O_2_ production, even when mitochondria were fueled with lactate (82, 83). Lactate fuels bioenergetics through its oxidation to pyruvate by mitochondrial lactate dehydrogenase (m-LDH), which is found in the matrix (84). Recently, we found KGDH is a major mH_2_O_2_ supplier during fatty acid oxidation as well, but only when the Krebs cycle was primed with malate (*unpublished*). In the context of oxidative eustress signaling, this puts KGDH in the ideal position for supplying mH_2_O_2_ for cell reprogramming. This is because KGDH is a point of convergence for carbon metabolized by several metabolic pathways that degrade amino acids, generate acetyl-CoA, and feed substrates into other parts of the Krebs cycle. These observations also point to a central role of the sustained and higher-than-normal rate for mH_2_O_2_ generation by KGDH in the onset of MAFLD and its further pathogenesis. Indeed, intrahepatic lipid accumulation and the augmentation of mH_2_O_2_ due to fatty acid oxidation have been implicated in the early onset of MAFLD (8, 9). The sustained overproduction of mH_2_O_2_ due to nutrient overavailability by PDH and KGDH likely occurs due to defects in mitochondrial bioenergetics and metabolic gridlock, which occurs because of poor diet or exposure to toxins leading to MAFLD (3, 85). Thus, oxidative eustress can be fueled through KGDH and PDH by the oxidation of several different nutrients but poor nutrition, toxins, and other factors that cause the overgeneration of mH_2_O_2_ by both enzymes can cause oxidative distress leading to MAFLD (Fig. 2).

## PDH and KGDH generate mH_2_O_2_ by RET from NADH

Purified KGDH was shown to generate H_2_O_2_ by RET from NADH 2 decades ago (28). As little as 1 μM NADH could induce H_2_O_2_ production (28). The NADH/NAD^+^ ratio also affected this production. Increasing NADH from 100 to 500 μM significantly augmented H_2_O_2_ generation by purified KGDH (28). NADH accumulation occurs when there are inherited or acquired defects in complex I activity, which correlates with the pathogenesis of MAFLD (86). Thus, inhibition of the ETC may drive high rates of mH_2_O_2_ by KGDH due to RET from NADH inducing oxidative distress (27). Follow up studies revealed purified PDH also generates H_2_O_2_ by RET (87). Notably, ≤1 μM NADH stimulated H_2_O_2_ generation by both purified PDH and KGDH. The rate of H_2_O_2_ production by PDH and KGDH reached its Vmax at 10 μM NADH (87). This is significant because the NADH/NAD^+^ ratio in the matrix is ∼8:1 with NADH occurring at ∼400 μM (88). Thus, production of mH_2_O_2_ by PDH and KGDH during RET from NADH is likely to occur *in vivo* and be amplified when complex I and the ETC are defective.

**Figure 3:**
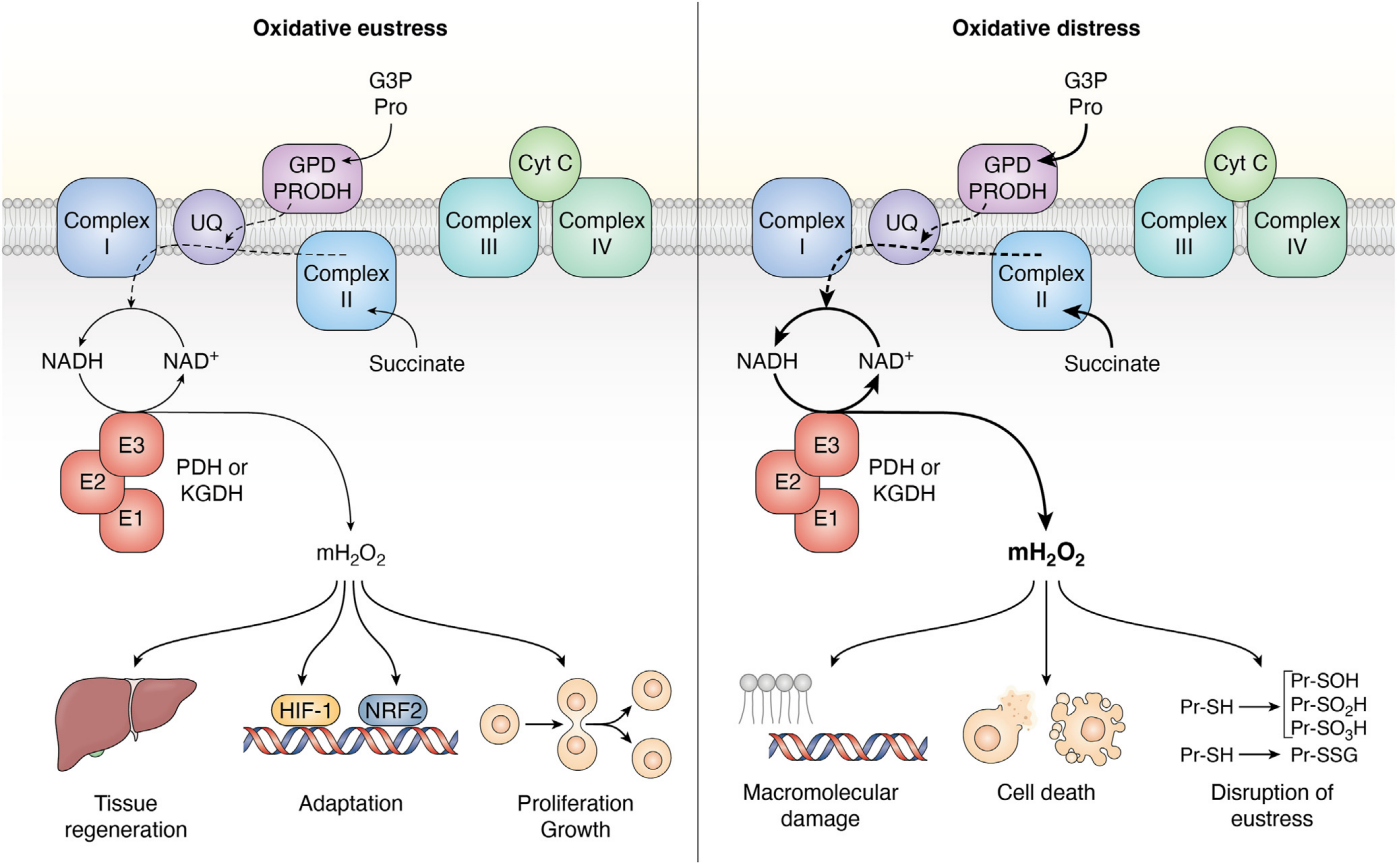
**Pyruvate dehydrogenase (PDH) and α-ketoglutarate dehydrogenase (KGDH) as major mitochondrial (mH**_**2**_**O**_**2**_**) sources during reverse electron transfer (RET) from the ubiquinone pool.** Under oxidative eustress conditions (*left*), electron flow from succinate, glycerol-3-phosphate, proline, and other sources generates NADH through proton return at complex I. Under these conditions, mitochondria are operating normally and the back fluxes in electrons are controlled. The NADH is then oxidized by PDH or KGDH (represented by the E1:E2:E3 subunits) and the electrons drive mH_2_O_2_ generation, which is transmitted into the cell to trigger proliferation and growth, cell adaptation, and tissue regeneration (*e.g.*, in the case of liver recovery from injury). Oxidative distress (*right*) is triggered by mitochondrial dysfunction and metabolic gridlock. This can result in the accumulation of ubiquinone metabolites (*e.g.*, succinate, glycerol-3-phosphate, proline), which drive the overgeneration of NADH by complex I through RET. This overloads PDH and KGDH with electrons resulting in the sustained overproduction of mH_2_O_2_. The resulting oxidative distress is related to the nonspecific and over-oxidation of protein cysteine thiols and the disruption of redox signaling circuits like protein S-glutathionylation (PSSG). The overgeneration of mH_2_O_2_ also triggers macromolecular damage and the induction of cell death. The figure was generated with Biorender Software (Agreement number: IC25TFIF36).

At one point, RET-driven mH_2_O_2_ production by PDH and KGDH could only be successfully measured *in vitro* using purified enzymes. Based on this, it was argued that such reactions likely do not occur *in vivo*. However, in a recent and important publication, it was revealed for the first time KGDH can generate mH_2_O_2_ by RET from NADH during the metabolism of ubiquinone-linked substrates like succinate or glycerol-3-phosphate *in vivo* (Fig. 3) (75). Using DLAT or DLD null rodents, the authors were able to show partial loss of KGDH significantly diminished mH_2_O_2_ generation by RET from succinate and glycerol-3-phosphate (75). In this model, succinate or glycerol-3-phosphate are oxidized by complex II and GPD, respectively, resulting in backflow through ubiquinone to complex I. The backflow triggers proton return through complex I, resulting in NADH production and the delivery of electrons to KGDH for mH_2_O_2_ generation (75). These findings are also highly significant because it reveals KGDH is an important source for sustained mH_2_O_2_ over production during pathogenesis. Ischemic-reperfusion injury to tissues is associated with a massive burst in mH_2_O_2_ production due to the overloading of the ETC with electrons from succinate and other ubiquinone-linked nutrients (*e.g.*, proline or glycerol-3-phosphate). Horvath *et al.* (75) show this source of mH_2_O_2_ could be KGDH. Additionally, this mechanism could play a significant role in the progression of MAFLD (Fig. 3). Succinate does accumulate in the diseased liver which can drive the over generation of mH_2_O_2_ through RET from complex II (89). Thus, under pathological conditions, oxidative distress can be triggered by RET from succinate through complex I and NADH to KGDH (and perhaps PDH). It is also likely that RET from the ETC to KGDH could trigger oxidative eustress pathways under normal cellular conditions (Fig. 3). Horvath *et al.* (75) did demonstrate that mitochondria from DLAT and DLD null rodents expressed less SOD2 and glutathione peroxidase-1 (GPX1), suggesting mH_2_O_2_ formed by RET to KGDH activates the nuclear factor erythroid 2–related factor 2 (Nrf2) pathway (Fig. 3). It needs to be emphasized that the experiments carried out by Horvath *et al.* (75) were done with isolated mitochondria, which lacks some relevance *in vivo*. It would be useful to follow up on these experiments with a cultured cell model containing a knockdown for PDH or KGDH. Additionally, such an approach could allow for the use of proteinaceous fluorescent mH_2_O_2_ sensors, like HyPER7 or Orp1, which can interrogate, in real time, subcellular H_2_O_2_ gradients (90). Such approaches can supply valuable information on the *in vivo* relevance of mH_2_O_2_ generation by RET through the ETC. Overall, RET from the ETC to PDH and KGDH could be the major mH_2_O_2_ source in oxidative eustress and distress.

## Redox modification of PDH and KGDH and metabolic rewiring for signaling

Inhibition of PDH and KGDH by oxidative distress was first documented in 1978 (91). This study found the treatment of isolated mitochondria with *t*-butyl hydroperoxide inhibited O_2_ consumption supported by pyruvate and α-ketoglutarate (91). Notably, this inhibition could be prevented with glutathione peroxidase (91). Later work discovered PDH and KGDH are inhibited by mH_2_O_2_ and lipid peroxidation end-products like 4-hydroxy-2-nonenal (HNE) (21, 27, 71, 92). Inhibition of KGDH by mH_2_O_2_ can be reversed by PSSG (71). Deactivation of KGDH by mH_2_O_2_ occurs through the oxidation of the vicinal thiols in the dihydrolipoamide to sulfenic acids (SOH) (31). During oxidative distress, the SOH can be modified further, undergoing more reactions with mH_2_O_2_ to form sulfinic (SO_2_H) and sulfonic acids (SO_3_H), irreversibly deactivating KGDH (25). The SOH can also be irreversibly modified with lipid peroxidation end products (31). It was found the PSSG formation in response to mH_2_O_2_-mediated reduced GSH pool oxidation reacted with the SOH in KGDH protecting the vicinal thiols in the lipoamide from further oxidative modifications (71). This modification was found to occur on the E2 subunit of PDH and KGDH, specifically the vicinal thiols of the dihydrolipoamide (31). Importantly, the glutathionylation could be reversed by glutaredoxin (71). Together, PSSG adduct formation in KGDH was uncovered to be a mechanism required to protect the enzyme from irreversible inactivation when cells are faced with temporary oxidative distress. Also, the addition and removal of a glutathionyl moiety to and from the E2 lipoamide was shown to modulate KGDH activity (31, 93). Glutathione addition to the E2 subunit blocks electron flow to the FAD, preventing NADH production (31, 93). The deglutathionylase activity of glutaredoxin reversed this modification, restoring electron flow through KGDH to the E3 subunit for NADH generation (31, 93). PDH was then discovered to be modulated by a similar reaction (78). Together, the reversible glutathionylation of PDH and KGDH is vital for protecting the enzyme complexes from irreversible oxidative deactivation but also serves as a means for regulating Krebs cycle flux through modulation of NADH generation.

**Figure 4:**
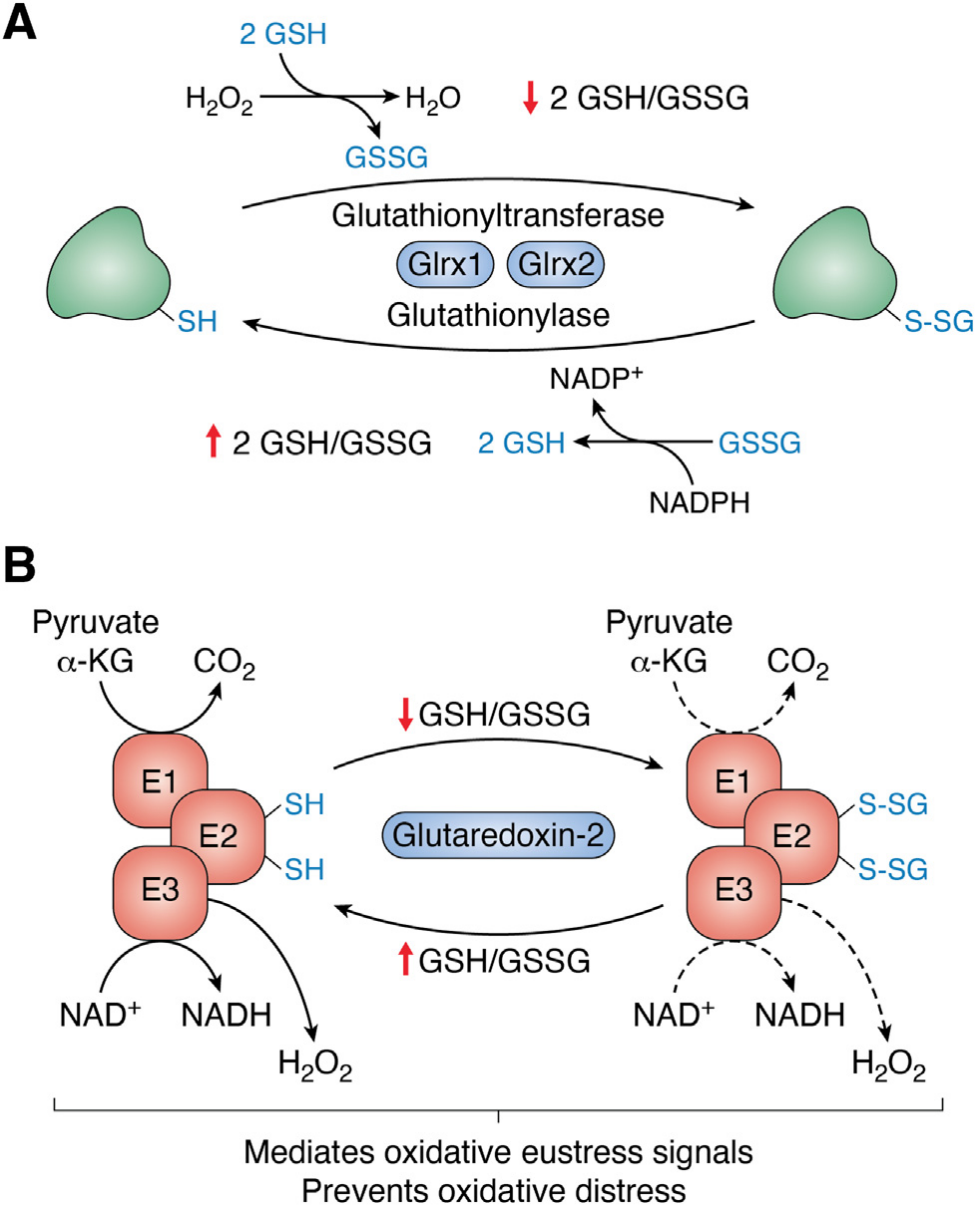
**The protein S-glutathionylation cycle and the control of mH**_**2**_**O**_**2**_**production by PDH and KGDH through the reversible addition and removalof GSH to and from the E2 subunit.***A*, reversible protein S-glutathionylation (PSSG) occurs in response to changes in mH_2_O_2_ availability and the generation of NADPH, which drive reduced glutathione (GSH) pool oxidation to glutathione disulfide (GSSG) and its reduction through peroxidases and reductases. The decrease in the GSH/GSSG ratio activates the glutathionyltransferase activity of glutaredoxin-2 (Glrx2), resulting in the formation of PSSG. Reduction of the GSH pool and the increase in GSH/GSSG induces Glrx2 deglutathionylase activity restoring protein function. In this way, glutathionylation regulates proteins in response to changes in redox tone. Figure was generated using Biorender Software (Agreement number: YM25TFIF86). *B*, the dihydrolipoamide of the E2 subunit of PDH and KGDH is targeted for reversible glutathionylation. This increases and decreases mH_2_O_2_ generation in response to changes in redox tone, protecting cells from oxidative distress but also simultaneously modulating cell oxidative eustress signals. Figure was generated using Biorender Software (Agreement number: XE25TFIFB9).

As highlighted earlier and in Figure 1*B*, DLD is the main site for mH_2_O_2_ generation in PDH and KGDH. It was hypothesized in 2015 that PSSG adduct formation was also required to serve as a tool for dampening mH_2_O_2_ generation by PDH and KGDH (94). This could be achieved through the blockage of electron flow through the E2 subunit by dihydrolipoamide modification (Fig. 4). In this proposed mechanism, GSH oxidation by a burst in mH_2_O_2_ generation results in glutathione disulfide (GSSG) formation and the blockage of PDH and KGDH by glutathionylation. The modification occurs on the E2 subunit preventing electron flow to DLD mitigating mH_2_O_2_ generation. Since 2015, empirical evidence has been collected demonstrating the E2 subunit modification with glutathionylation catalysts like diamide or disulfiram or following GSSG formation inhibits mH_2_O_2_ generation (Fig. 4) (50, 95, 96). Notably, these reactions were found to be mediated by glutredoxin-2 (Glrx2), which drives glutathionylation and deglutathionylation reactions in response to GSH pool oxidation and reduction in the matrix of mitochondria (Fig. 4) (97). The induction of PSNO with S-nitroso-glutathione (GSNO) was also recently found to elicit the same effect on mH_2_O_2_ on PDH and KGDH, although the former was documented to be more resistant to modification (98).

In the context of oxidative eustress signaling, the redox modification of PDH and KGDH by reversible PSSG or PSNO can induce metabolic rewiring for adaptive signaling and intercellular communication. For PSSG, the reversible modulation of mH_2_O_2_ protects from oxidative distress but also simultaneously modulates eustress signals for cellular adaptation and proliferation (Fig. 4). A recent study confirmed PDH undergoes glutathionylation on the E2 subunit, which modulates mH_2_O_2_ generation in macrophages (32). Interestingly, the authors also show this occurs in response to the induction of inflammatory signaling cascades, implicating reversible glutathionylation of PDH in the immunomodulatory response (32). Recent work has also identified a novel mechanism where reversible cysteine nitrosylation (PSNO) of PDH and KGDH rewires the Krebs cycle to modulate the availabilities of itaconate, 2-hydroxyglutarate, succinate, and mH_2_O_2_, which serve as powerful interorganelle and intercellular signaling molecules. This can have profound physiological effects such as in the modulation of immune cell function. Induction of nitric oxide generation in macrophages by lipopolysaccharide (LPS) or interferon-γ triggers the changes in the availability of these immunomodulatory metabolites through the PSNO modification of the E2 subunit of PDH and KGDH (33). Immune cell activation through this rewiring begins with the generation of itaconate by aconitate decarboxylase (99). The itaconate is generated from aconitate in the Krebs cycle and released into the extracellular milieu where it reacts with OXGR1, a GPR that was first found to bind α-ketoglutarate (33). Additionally, itaconate inhibits complex II, prompting succinate accumulation, another important intercellular signaling molecule. Together, itaconate and succinate orchestrate the immune response through stabilization of transcription factors (*e.g.*, hypoxia-inducible factor-1α (HIF-1α) and NRF2) and transmitting signals through OXGR1 and SUCNR1 (33, 100, 101). The strong inhibition of PDH and KGDH by PSNO is a feedback loop that limits itaconate and succinate generation through Krebs cycle deactivation (33). The generation of mH_2_O_2_ by PDH and KGDH would reinforce these signaling effects since it has the same targets for immune cell activation as itaconate and succinate (98). The PSNO modification of PDH and KGDH would also dampen the mH_2_O_2_ oxidative eustress signals emanating from mitochondria as well (98). It is anticipated that PSSG also modulates the availability of key immunomodulatory metabolites like itaconate and succinate. In the context of oxidative distress, nitro-oxidative stress that leads to the prolonged deactivation of mitochondrial proteins by PSNO has been linked to metabolic disorders like MAFLD (102). ETC and Krebs cycle components display inactivation due to PSNO adduct formation in fatty liver disease (103). As outlined earlier, reversible PSNO can be vital for oxidative eustress signaling but it must also be anticipated that defects in this pathway and/or oxidative distress will disrupt liver cell metabolism leading to pathogenesis. PDH and KGDH are deactivated in MAFLD and restoration of their activities is a suggested therapeutic target for the treatment of liver diseases (14). Notably, lipoic acid supplementation does curtail PSNO adduct formation and the inhibition of PDH and KGDH (104). Overall, the protein S-glutathionylation and nitrosylation of the E2 subunit of PDH and KGDH may be integral for metabolic rewiring in cell signaling and defects in this regulatory pathway can lead to pathogenesis.

## Sex and diet effects on the S-glutathionylation and S-nitrosylation of PDH and KGDH in the pathogenesis of MAFLD

PSSG has profound regulatory effects on mitochondria (105, 106). There are ∼2200 targets in mammalian cells, with many found in mitochondria (107). PSSG in response to the oxidation and reduction of the GSH pool modulates the rate of mH_2_O_2_ production by complexes, I, II, and III, KGDH, PDH, and dihydroorotate dehydrogenase (DHODH) in mitochondria and xanthine oxidoreductase in rat liver cytoplasm, suggesting it’s a cell wide dampener for oxidative eustress signals (97). In the context of PDH and KGDH, both enzymes account for nearly 50% of the total mH_2_O_2_ generation in liver mitochondria, making PSSG an important regulator of oxidative eustress signals from both enzymes. Importantly, there are sex dimorphic effects related to the PSSG and PSNO of PDH and KGDH and defects in these pathways have been linked to the pathogenesis of MAFLD due to a poor diet. Feeding rodents a high-fat diet (HFD) induces simple steatosis, weight gain, and hypertrophying of adipose in male mice which correlates with the increased PSNO modification and inhibition of PDH and KGDH (98). Interestingly, female rodents were highly resistant to the PSNO-mediated inhibition of PDH and KGDH in liver mitochondria (98). Similar findings have been made with PSSG. PDH and KGDH are more resistant to PSSG modifications in liver mitochondria collected from female mice (80). Importantly, we had identified Glrx2 as a major mediator for these reactions in liver mitochondria, but only in males, not females (80). The sex dimorphic effect in PSSG adduct formation in liver mitochondria was related to differences in redox tone and mH_2_O_2_ buffering capacity (80, 108). Indeed, liver mitochondria from female rodents and humans have greater antioxidant capacity and redox tone, better mitochondrial coupling between nutrient oxidation and ATP production, and increased proton leaks, which reduces protonic backpressure to limit ROS production (11, 108, 109).

The sex dimorphic effect in PSSG adduct generation on PDH and KGDH (and other mitochondrial proteins) could account for, to some degree, sex dimorphisms in MAFLD progression. MAFLD induced by poor diet (*e.g.*, HFD), protein deficiency or changes in protein diet composition, or exposure to xenobiotics like paracetamol is more prevalent in male rodents when compared to females (1, 110, 111). This has been related to estrogen signaling and the prevention of mitochondrial dysfunction and oxidative distress in hepatic mitochondria (112). Notably, the sex dimorphic differences in GSH availability and redox tone may also impact the role of PSSG reactions in the prevention of MAFLD. Knockout of the Glrx2 gene in male mice augments paracetamol hepatotoxicity through the induction of mitochondrial dysfunction by defects in PSSG formation (113). This correlates with intrahepatic lipid accumulation, oxidative distress, apoptosis inducible factor activation, and cell death (113). Similar findings have been generated with Glrx2 null rodents fed a high fat diet. Deleting the Glrx2 gene in male mice induces mitochondrial dysfunction and aberrant increases in PSSG formation following exposure to a high fat diet (114, 115). This was accompanied by the induction of steatosis and liver dysfunction (115). Notably, Grlx2 null female mice exhibit no increased sensitivity towards HFD-induced MAFLD, and its loss did not alter the efficiency of OxPhos or redox tone when compared to wild-type littermates fed the same diet (108). PDH and KGDH in Glrx2-null mice display increased glutathionylation and deactivation in livers (96). This is associated with decreased mH_2_O_2_ generation by PDH and KGDH but is also associated with a total increase in mitochondrial PSSG adducts and oxidative distress (96). It was recently shown aberrant PSSG formation can induce the overproduction of mH_2_O_2_ generation by the ETC during the oxidation of succinate, proline, or glycerol-3-phosphate (116). Thus, loss of Glrx2 results in disruption of PDH and KGDH and overall metabolism in hepatocytes culminating with lipid accumulation, metabolic gridlock, and MAFLD development, but only in male mice. Surprisingly, male mice heterozygous for Glrx2 are fully protected from HFD-induced obesity and MAFLD (117). Wild-type male littermates develop steatosis, insulin resistance, adipose hypertrophying, and hepatocytes are depleted of glycogen (117). This is prevented in mice where one of the two Glrx2 genes was deleted (117). The positive effects related to the loss of one of the two Glrx2 genes is related to induction of oxidative eustress signals and the bolstering of antioxidant defenses (117). Ablation of Glrx1, which is the Glrx2 isozyme localized in the cytoplasm, also elicits hepatocellular damage and dyslipidemia in response to a HFD (118). Loss of Glrx1 sensitizes male mice, but not female ones, towards HFD induced oxidative distress and intrahepatic lipid accumulation (119). Finally, Glrx1-null male mice develop hepatic inflammation and steatosis and gut dysbiosis in response to a diet rich in processed meat (120). These effects are related to the induction of oxidative distress in hepatocytes.

## Conclusion

MAFLD and its more severe forms are surging due to the global rise in obesity and metabolic syndrome. Poor nutrition and exposure to xenobiotics also drive the pathogenesis of MAFLD. Its manifestation is difficult to detect until the onset of cirrhosis and hepatocellular carcinoma. Defects in mitochondrial metabolism and redox tone are common features in MAFLD progression. This has led to the development of interest in targeting mitochondria and its redox environment in the treatment of early steatosis and its more severe forms. Here, we have elaborated on the central role of PDH and KGDH in mediating oxidative eustress signals, which can be targeted to facilitate liver recovery in response to hepatic injury. Dysfunction in PDH and KGDH redox metabolism can have the opposite effect, eliciting oxidative distress which is characterized by dysfunctional mH_2_O_2_ signals, cell damage, and the induction of death. PDH and KGDH are important mH_2_O_2_ sources and sites for redox regulation, making both enzymes important targets for metabolic rewiring for the induction of interorganelle and intercellular signaling. Collectively, the targeted manipulation of PDH and KGDH redox metabolism can serve as a valuable means in treating the global rise in MAFLD.

## Conflict of interest

The authors declare that they have no known competing financial interests or personal relationships that could have appeared to influence the work reported in this paper.
